# Pathological responses and survival outcomes in patients with locally advanced breast cancer after neoadjuvant chemotherapy: a single-institute experience

**DOI:** 10.1186/s43046-021-00096-y

**Published:** 2021-12-14

**Authors:** Kyrillus S. Shohdy, Doaa S. Almeldin, Madonna A. Fekry, Mahmoud A. Ismail, Nedal A. AboElmaaref, Esraa G. ElSadany, Baher M. Hamza, Fatma H. El-Shorbagy, Ahmad S. Ali, Hanaa Attia, Loay Kassem

**Affiliations:** 1 Department of Clinical Oncology, Kasr Alainy School of Medicine, Cairo University, Cairo, Egypt; 2 Division of Hematology and Medical Oncology, Department of Medicine, Weill Cornell Medical College, New York, NY USA; 3 Department of Radiation Oncology, Rutgers Cancer Institute of New Jersey, New Brunswick, NJ USA; 4 Faculty of Pharmacy, Modern Sciences and Arts (MSA) University, Cairo, Egypt; 5 Department of Obstetrics and Gynecology, Kasr Alainy School of Medicine, Cairo University, Cairo, Egypt; 6 Kasr Alainy School of Medicine, Cairo University, Cairo, Egypt

**Keywords:** Breast cancer, Pathological complete response, Survival outcomes

## Abstract

**Background:**

Pathological complete response (pCR) is a surrogate for the efficacy of neoadjuvant chemotherapy (NCT) in locally advanced breast cancer (LABC). We analyzed the predictive clinical factors for pathological responses and survival outcomes in a cohort of Egyptian patients.

**Methods:**

We evaluated the medical records of patients with breast cancer who received NCT in our academic institute. Survival curves were estimated with the Kaplan-Meier method. Cox proportional models were used for multiple regression analysis.

**Results:**

Our cohort included 368 patients with a median age of 48 years (range 21–70). The median follow-up time was 3 years. The clinical tumor stage (T3–4) represented 58%, with 80% having positive axillary nodes. The luminal subgroup prevailed by 68%. The objective response rate (ORR) reached 78%, and 16% of patients achieved pCR. The clinical node stage and optimal chemotherapy were associated with higher ORR (*p* = 0.035 and *p* = 0.001, respectively). Predictors of pCR were clinical T-stage (*p* = 0.026), high Ki-67 index > 20 (*p* = 0.05), and receiving optimal chemotherapy (*p* = 0.014). The estimated 3-year disease free-survival (DFS) was 53%. Receptor status, achieving ORR, and pCR were associated with better DFS with hazard ratios of 0.56, *p* = 0.008; 0.38, *p* = 0.04; and 0.28, *p* = 0.007, respectively.

**Conclusions:**

Luminal tumors still draw benefit from neoadjuvant chemotherapy in terms of clinical response and breast conservative surgery. Treatment escalation to those who did not achieve pCR requires more investigation, given a higher recurrence rate in real-world experience.

## Background

Breast cancer is the commonest female malignancy, with 276,480 new cases and 42,170 deaths expected in the USA in 2020 [1]. In Egypt, breast cancer represented 32% of newly diagnosed female cancers [2, 3] and ranked as the first cause of female cancer deaths in the World Health Organization’s report 2014 [2]. A major proportion of our patients present with locally advanced breast cancer (LABC). Neoadjuvant chemotherapy (NCT) is increasingly adopted by our breast cancer multidisciplinary teams (MDTs). Besides its usefulness to downstage inoperable LABC and increased rates of conservative breast surgery in the operable cases, NCT proved to be an excellent platform for studying different prognostic factors for long-term outcomes, especially pathological complete response (pCR) [4]. In this cohort of Egyptian breast patients treated at a single academic institute, we looked for clinicopathological criteria of our LABC patients to analyze rates of pCR across different subtypes, predictors of these rates, and association with long-term disease outcomes.

## Methods

This retrospective cohort study included patients with histologically proven breast cancer who received neoadjuvant chemotherapy at who received neoadjuvant chemotherapy at Cairo University Hospitals between 2010 and 2015. The study was approved by IRB (Research Ethics Committee of Cairo University issued in April 2018). The data extracted from patient medical records included demographics, clinical stage, MDT treatment plans, pathological criteria for cores, and surgical specimens. Our surgical approach includes post-NCT axillary lymph node dissection (ALND) irrespective of treatment response and sentinel lymph node dissection (SLND) for clinically negative nodes. Outcome data were collected, including local, distant relapse, and decease status, to calculate different clinical outcomes survival endpoints. The 7th edition of the American Joint Committee on Cancer (AJCC) staging systems for breast cancer was used in this analysis.

### Statistical analysis

The analysis was conducted by STATA 14.2 (TX, USA); *p*-value is considered significant if < 0.05. Statistical analysis used the chi-square test and logistic regression for dichotomous variables and *t*-test for continuous variables. Pearson’s chi-square test was used to examine whether the distribution of clinical and pathologic factors was similar among patients from different subtypes. The chi-square test or Fisher’s exact test was also used to examine the associations between different clinical, pathologic factors and pCR to neoadjuvant therapy. Multiple analyses using Cox regression models included baseline factors (age, T N stage, histological type, tumor grade, and subtype) and pathological complete response status. We excluded patients with missing variables from the multiple regression analyses. Kaplan-Meier curves were used to estimate disease-free survival (DFS) and overall survival (OS). A log-rank test was used to compare the survival curves.

## Results

### Baseline clinicopathological criteria

Herein, we report on a cohort of 368 Egyptian LABC female patients. The median age at diagnosis was 48 years (range 21–70). The majority were premenopausal 206 (53%). The mean tumor size within the cohort was 5.5 cm (2–11 cm). Clinically, cT1–2 disease represented 42%, cT3–4 58%, and node-positive were 80% at the baseline clinical assessment that included physical examination and diagnostic sonomammography; 89.5% of the cores were invasive ductal carcinoma (IDC), and 4.4% were invasive lobular carcinoma (ILC). Hormone receptor-positive subtype (HR-positive) was the predominant in 68%, followed by HER2-positive (17%) and triple-negative (15%) subtypes. Other baseline clinicopathological criteria are outlined in Table 1.

**Table 1:** Clinical and histopathological criteria of the study cohort

Parameter	Frequency (*N* = 368)	Percent (%)
Age, median (range)	48 (21–72)	
Menopausal status		
Premenopausal	206	60
Postmenopausal	157	40
Clinical T stage		
T1	19	8.68
T2	74	33.8
T3	56	25.6
T4	70	32
Tx	48	13
Unknown	101	27.4
Clinical N stage		
N0	36	20
N1	88	49
N2	35	19.4
N3	21	11.7
Nx	15	4.7
Unknown	173	47
Histopathology of cores		
IDC	329	89.4
ILC	16	4.4
Unknown	23	6.2
Lymphovascular invasion		
Present	45	12.2
Absent	79	21.5
Unknown	244	66.3
Grade		
1	0	0
2	227	88.7
3	28	11.3
Unknown	112	30.4
Subtypes		
HR+/Her2−	164	53.8
HR+/Her2+	46	15.1
Her2-enriched	45	15.1
TNBC	50	16.4
Unknown	63	17.1

*IDC* invasive ductal carcinoma, *ILC* invasive lobular carcinoma, *HR* hormone receptor, *HER2* human epidermal receptor 2, *TNBC* triple-negative breast cancer

### Treatment regimens and surgical interventions

All medically fit patients received anthracycline/taxanes containing regimen, consisting of doxorubicin 60 mg/m^2^ and cyclophosphamide 600 mg/m^2^ (AC) once every 3 weeks for four cycles followed by paclitaxel (T) 80 mg/m^2^ weekly for 12 cycles. Most of the patients with HER2-positive disease received trastuzumab concurrently with taxanes (8 mg/kg loading dose and then 6 mg/kg every 3 weeks). Following NCT, all patients were discussed within MDT for surgical interventions, either mastectomy or breast conservative surgery.

### Clinical response

The objective response rate (ORR) to NCT (defined as more than 50% decrease in the largest tumor dimension by clinical examination) in 122 evaluable patients was 78% (95/122). We limited this analysis to patients with serial clinical tumor size reported numerically in the visits during neoadjuvant treatment; 25.5% (31/122) of patients achieved a clinical complete response (CR), 52.5% (64/122) had a partial response (PR), 20.5% (25/122) with stable disease (SD), and two patients developed disease progression (DP).

ORR was significantly higher in patients who completed an optimal course of NCT (≥ 6 cycles) compared to receiving sub-optimal course, i.e., < 6 cycles (62% vs. 15%, *p* = 0.001). The clinical nodal stage was significantly associated with ORR, specifically node-positive patients achieving higher ORR compared to node-negative patients in the simple regression analysis (48% vs. 10%, *p* = 0.035). No significant difference in ORR was observed according to HR status (41% vs. 21%, *p* = 0.111). There was a numerically higher ORR in younger patients, i.e., < 50 years (81.5% vs. 73.2%, *p* = 0.2).

### Pathological response

Surgical specimens of 280 patients were evaluable for pathologic response assessment. The median pathologic tumor size was 3 cm (range 0–13). The median number of dissected lymph nodes was 14 (2–43), and the median number of the positive lymph nodes was 2 (0–37).

Residual tumor tissue of grade 2 was reported in 36% of specimens with adverse pathological features, including lymphovascular invasion (LVI) reported in 32% and extracapsular extension reported in 13% of the surgical specimens. A total of 44 patients (15.7%) achieved pCR in both breast and axilla (ypT0-is, ypN0), and 55 patients (20%) achieved pCR only in the breast (ypT0). Postoperative pathological outcomes are summarized in Table 2.

**Table 2:** Pathological responses after neoadjuvant chemotherapy

Parameter	Frequency (*N* = 280)	Percent (%)
Pathologic T stage		
0	55	19.6
1	56	20
2	86	30.7
3	40	14.2
4	39	14.2
yTx	4	1.4
Pathologic N stage		
0	92	33.7
1	84	30.8
2	64	23.5
3	33	12.1
yNx	7	
pCR (T0/Tis)		
No pCR	175	62.5
Breast and axilla	44	15.7
Breast only	12	4.3
Axilla only	49	17.5
Adverse features		
LVI	89	31.7
ECE	37	13.2

*pCR* pathological complete response, *LVI* lymphovascular invasion, *ECE* extracapsular extension

### Predictive factors for pCR

The pCR was highly associated with receiving optimal chemotherapy course (≥ 6 cycles) compared to the suboptimal course (22.2% vs. 8.6%, *p* = 0.014) (see Table 3). Patients with clinical tumor stages cT1–2 achieved pCR significantly more than tumor stages cT3–4 (27.1% vs. 13.4%, *p* = 0.026). High Ki-67 index > 20% correlated with higher incidence of pCR (41.18% vs. 22.83%, *p* = 0.05). In our institute, we use the 20% cutoff to define luminal tumors with a higher proliferative index. The HR-positive/HER2-negative patients were less likely to achieve pCR compared to HR-negative subtypes (13% vs. 21.5%, *p* = 0.07). Achieving pCR was associated with performing breast conservative surgery (BCS) more than modified radical mastectomy (MRM) (26.92% vs. 8.07%, *p* < 0.001). The age, grade, tumor size, nodal stage, HR, and HER2 status were not associated with a significant difference in pCR in logistic regression analysis outlined in Table 4.

**Table 3:** Association of clinicopathological characteristics and pCR

Variables	pCR events (%)	*p*-value*
Age		
≤ 50 years	23/151 (15.23)	0.789
> 50 years	21/128 (16.41)	
cT1–2	21/72 (27.14)	**0.026**
cT3–4	14/100 (13.40)	
cN0	9/25 (25.7)	0.111
cN1–3	26/123 (21.1)	
HR+/HER2−	17/131 (13)	0.069
Other subtypes	25/116 (21.55)	
Grade		
G2	28/173 (16.18)	0.136
G3	6/21 (28.57)	
LVI		
No	17/74 (22.97)	0.07
Yes	3/35 (8.57)	
Ki-67		
Low index	21/92 (22.83)	**0.05**
High index	14/35 (41.18)	
Neoadjuvant chemotherapy		**0.014**
Optimal ≥ 6 cycles	34/153 (22.22)	
Suboptimal < 6 cycles	6/70 (8.57)	

*p-values were computed using the chi-square test

**Table 4:** Simple and multiple logistic regression analysis of predictors of pathologic complete response (pCR)

	Odds ratio	*p-value*	95% confidence interval	
pCR (simple regression)				
Age group (≤ 50 vs. > 50)	1.09	0.79	0.57	2.08
cT1, 2 vs. cT3, 4	0.40	**0.017**	0.18	0.85
N0 vs. N+	0.48	0.116	0.20	1.20
HR (positive)	0.74	0.378	0.38	1.44
HER2 (positive)	1.21	0.624	0.57	2.58
Optimal chemotherapy (yes)	3.05	**0.018**	1.22	7.64
KI-67 (high)	2.25	**0.056**	0.98	5.19
pCR (multiple regression)				
Age group (≤ 50 vs. > 50)*	1.09	0.854	0.43	2.77
cT1, 2 vs. cT3, 4*	0.28	**0.008**	0.11	0.71
N0 vs. N+*	0.59	0.4	0.17	2.01
HR (positive)*	0.69	0.435	0.27	1.78
Optimal chemotherapy (yes)*	1.49	**0.016**	1.08	2.06

*These variables were included in the same multiple logistic regression model

### Survival analysis

**Fig. 1:**
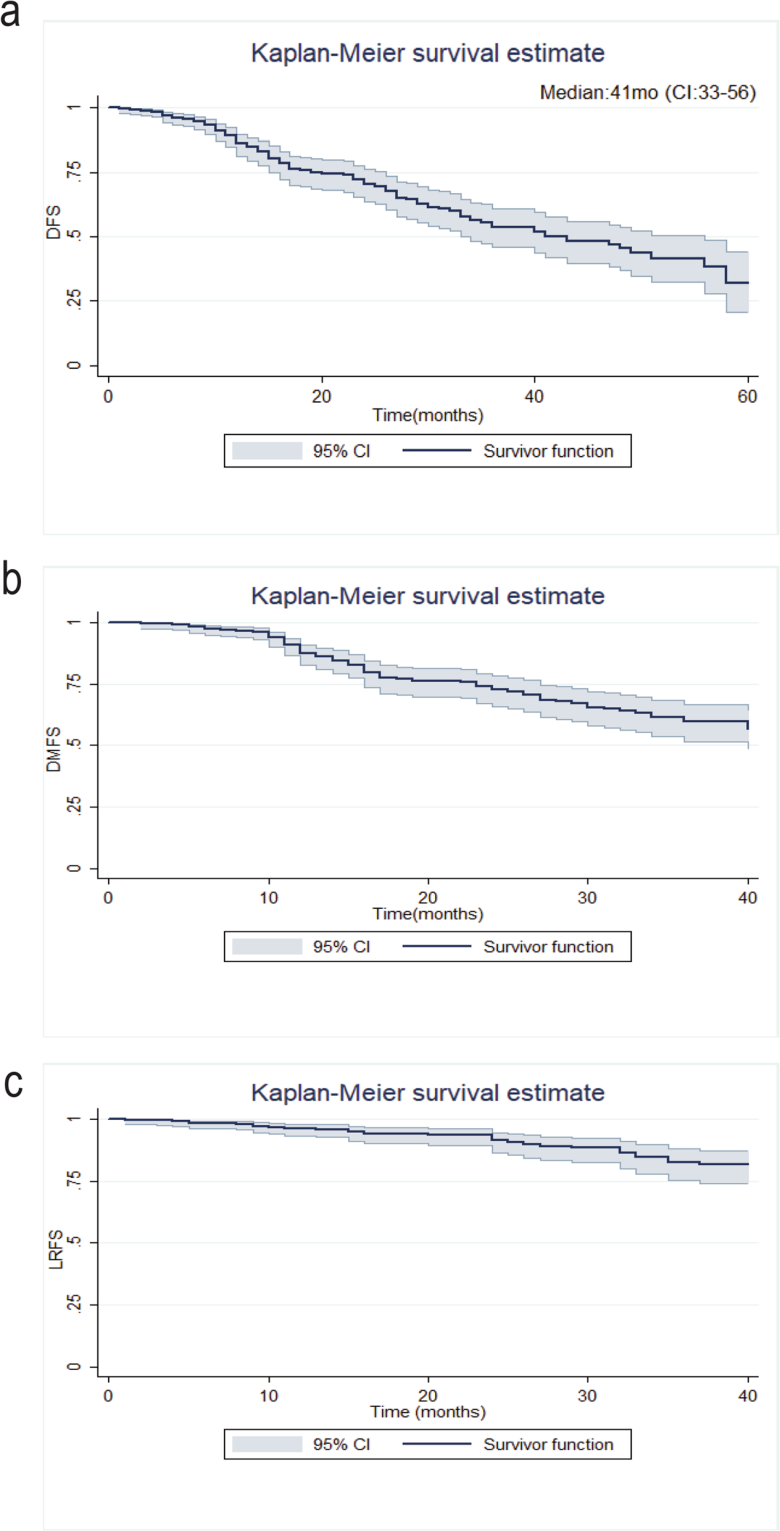
Kaplan-Meier survival curves of **a** disease-free survival (DFS), **b** distant metastasis-free survival, and **c** local relapse-free survival of the study population

Out of the 368 patients, 110 (30%) developed disease recurrence within the study follow-up time. Local recurrence occurred in 36 patients (32.7%) and distant recurrence in 95 patients (86%). Meanwhile, 21 patients (19%) had a simultaneous local and distant relapse. The estimated 3-year and 5-year disease-free survival (DFS) was 53% and 32%, respectively. The estimated 3-year local recurrence-free survival (LRFS) was 60.9%, and distant metastasis-free survival (DMFS) was 82.4% (Fig. 1).

**Fig. 2:**
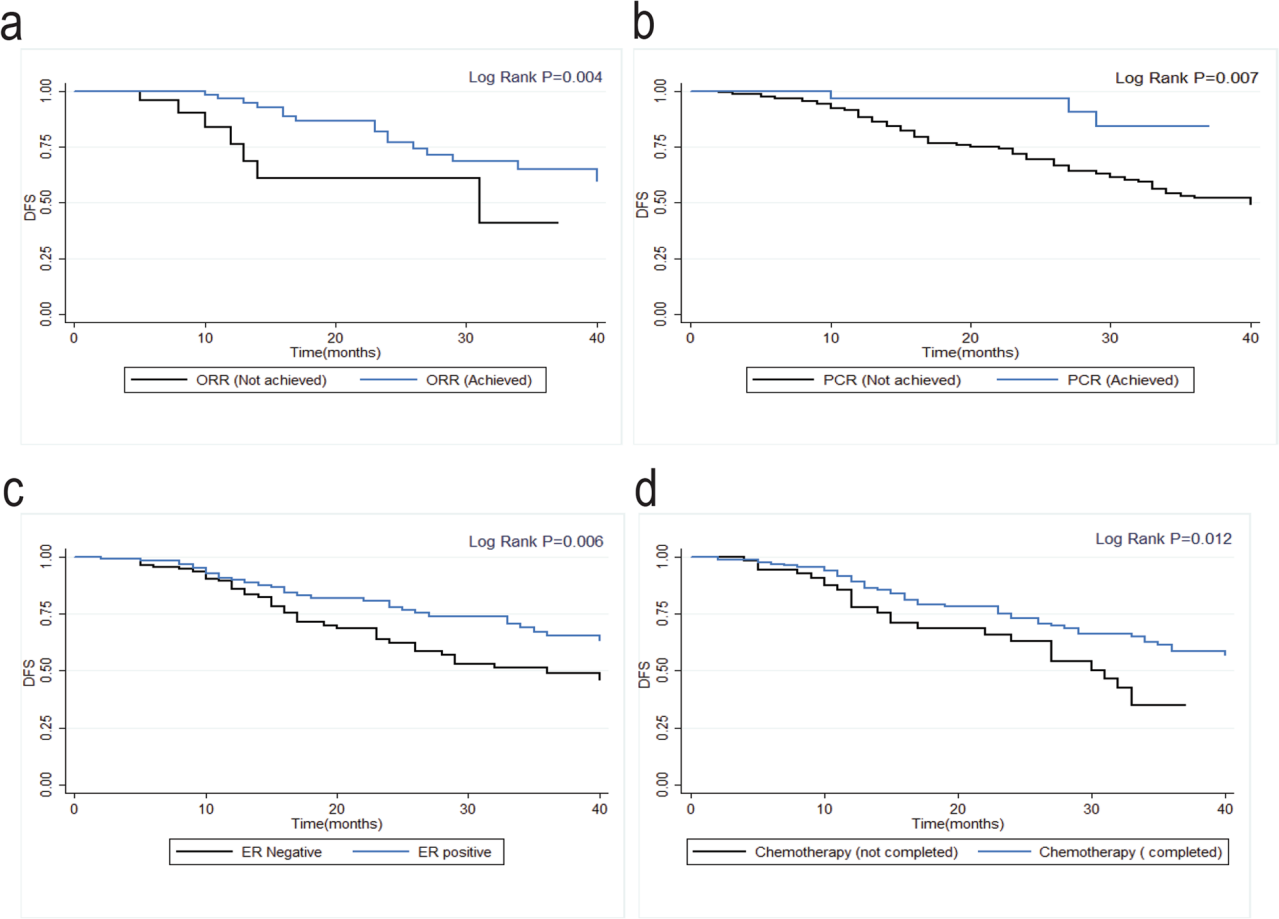
Survival curves of significant predictors for disease-free Survival (DFS) with log-rank test *p-*values

**Fig. 3:**
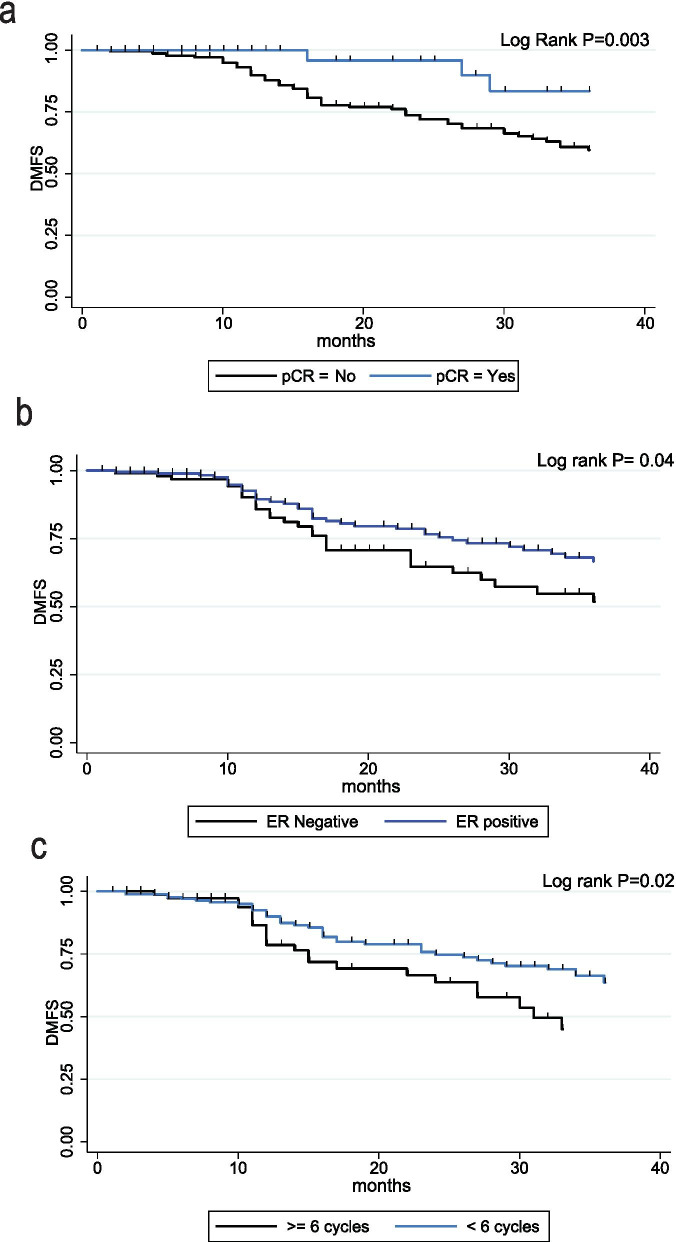
Survival curves of significant predictors for distant metastasis-free survival (DMFS) with log-rank test *p*-values

Significant predictors of DFS were HR status (hazard ratio (HR) 0.56; 95% CI 0.37–0.86, *p* =.008), ORR (HR 0.38, 95% CI 0.15–0.95, *p* = 0.04), receiving optimal chemotherapy cycles (HR 0.56, 95% CI 0.35–0.87; *p =* .012), and achieving pCR (HR 0.28, 95% CI 0.11–0.73, *p* = 0.007) (Fig. 2). In a multiple regression analysis adjusted for clinicopathologic parameters, pCR was an independent predictor of DFS (HR 0.35, 95% CI 0.1–0.8, *p* = .02). Simple regression analyses and multiple analyses are summarized in Table 5. The significant predictors of DMFS were optimal chemotherapy cycles (HR 0.57, 95% CI 0.35–0.9, *p =* .02) and achieving pCR (HR 0.34, 95% CI 0.1–0.8, *p* = 0.02) in both simple and multiple analyses (see Table 6 and Fig. 3).

**Table 5:** Univariate and multiple Cox regression analyses of predictors of disease-free survival

	Hazard ratio	*p*-value	95% confidence interval	
DFS (simple)				
Age group (≤ 50 vs. > 50)	0.694	**0.075**	0.464	1.03
HR (positive)	0.561	**0.008**	0.37	0.86
cT1, 2 vs. c T3, 4	0.0807	0.481	0.44	1.46
N+ vs. N0	1.97	0.22	0.60	6.40
ORR (yes)	0.38	**0.04**	0.02	0.96
pCR (yes)	0.288	**0.007**	0.12	0.71
HER2 (positive)	1.0404	0.9	0.56	1.93
Optimal chemotherapy (yes)	0.5613	**0.012**	0.36	0.89
DFS (multiple)				
Age group (< 50 vs. > 50)*	0.42	**0.004**	0.23	0.76
HR (positive)*	0.46	**0.004**	0.27	0.78
pCR (yes)*	0.30	**0.022**	0.11	0.84
Optimal chemotherapy (yes)*	0.52	**0.022**	0.29	0.91

Significant *p-*value in bold

*DFS* disease-free survival, *HR* hormone receptor, *ORR* objective response rate, *pCR* pathological complete response

*These variables were included in the same multiple Cox regression model

**Table 6:** Simple and multiple Cox regression analyses of predictors of distant metastasis-free survival (DMFS)

	Hazard ratio	*p-*value	95% confidence interval	
DMFS (simple)				
Age group (≤ 50 vs. > 50)	0.70	0.113	0.46	1.09
HR (positive)	0.65	0.065	0.41	1.03
cT1, 2 vs. c T3, 4	0.74	0.372	0.39	1.43
N0 vs. N+	2.47	0.218	0.58	10.47
ORR (yes)	0.33	**0.02**	0.13	0.84
PCR (yes)	0.34	**0.021**	0.14	0.85
HER2 (positive)	0.89	0.727	0.45	1.75
Optimal chemotherapy (yes)	0.58	**0.024**	0.36	0.93
DMFS (multiple)				
Age group (≤ 50 vs. > 50)*	0.45	**0.012**	0.24	0.84
HR (positive)*	0.51	**0.022**	0.29	0.91
pCR (yes)*	0.34	**0.044**	0.12	0.97
Optimal chemotherapy (yes)*	0.51	**0.027**	0.28	0.93

Significant *p-*value in bold

*HR* hormone receptor, *ORR* objective response rate, *pCR* pathological complete response

*These variables were included in the same multiple Cox regression model

In the current cohort, 20% of patients had complete adjuvant endocrinal therapy data, for which 50% were on luteinizing hormone-releasing hormone (LHRH) agonist in addition to oral endocrinal therapy (tamoxifen or aromatase inhibitors). Due to the insufficient long-term data, this factor was not included in the regression analysis of survival outcomes.

## Discussion

This study frames an Egyptian experience with neoadjuvant systemic treatment for LABC represented by this cohort treated at a single-academic institute. It sheds light on our unique clinicopathological criteria of this advanced disease stage and explores their prognostic value. The identified predictive factors shall serve as informative tools for oncologists in a similar setting to tailor the management and follow-up plan.

We frequently encounter younger female patients with LABC. The median age of our cohort is 48 years, which is 14 years younger than the Western population median (62 years) [5]. This is a consistent observation with other national data [5, 6]. For example, in a study comparing Egypt’s Gharbia Cancer Registry (GCR) and the United States Surveillance, Epidemiology, and End Results (SEER) registries, Egyptian GCR cases were, on average, over 10 years younger than US SEER cases, with nearly 19% of GCR cases ≤ 40 years of age as compared to only 6% of US SEER cases [5].

High-risk biological profiles were evident in our cohort, with more than 50% having either HER2-positive or triple-negative breast cancer (TNBC) subtypes. Although some of these breast tumors were amenable for upfront BCS, our tumor board prefers to start neoadjuvant therapy for these higher-risk features. Concerning smaller breast sizes, BCS was sometimes very challenging in larger T2–3 cases. T4 patients represented 32% and were indicated for upfront mastectomy. All the above mandates special clinical practice implications like tendency to treatment escalation and increased need for fertility preservation counseling.

Our cohort comprised relatively more locally advanced cases compared to Western data [5]. Clinical stages IIIA and IIIB constituted 58% of our cohort, with 80% having node-positive disease compared to 37% in EBCTCG data [4] and 46% in Cortazar et al. [7]. Also, the incidence of luminal subtype was relatively higher than other reported cohorts (68%), with a relatively lower prevalence of TNBC (16.4%). This is in concordance with previous reports from Egypt [8, 9], suggesting a profile of locally advanced breast cancer in Egyptian patients with younger age, more luminal disease, and more advanced clinical T and N stages. This, in part, explains the relatively higher rates of clinical response despite similar pCR rates compared to international studies [10–12].

The clear effect of optimal chemotherapy course on the ORR, pCR, and survival outcomes confirms the importance of patient counseling to comply with pre-planned treatment courses. This is crucial in limited-resource settings when completing chemotherapy cycles could sometimes be overlooked if a remarkable response is achieved during NCT. We noticed that this practice was evident outside specialized breast cancer centers when patients were then referred to us for operative/postoperative management.

Achieving pCR in our cohort was significantly associated with survival outcomes. This comes in concordance with a recently published large meta-analysis by Laura et al., including more than 27,000 patients who were evaluated from 52 studies on NCT. Patients who had pCR, as compared to the absence of pCR, had significantly better event-free survival and overall survival (HR 0.31, 95% PI 0.24–0.39, *n* = 26,378, and HR 0.22, 95% PI 0.15–0.30, *n* = 23,329, respectively) [13]. pCR remained associated with significantly improved event-free survival (EFS) even with the absence of adjuvant chemotherapy (HR 0.36, 95% PI 0.27–0.54, *n* = 18,462).

Accumulating evidence has pointed to the poor outcomes observed in patients who failed to achieve pCR after NCT and the need for treatment intensification accordingly. For example, adjuvant capecitabine improved both DFS and OS when combined with standard adjuvant therapy for HER2-negative patients with residual invasive disease [14]. In addition, Trastuzumab emtansine (T-DM1) halved the risk of recurrence or death compared to adjuvant trastuzumab in HER2-positive patients who failed to attain pCR after standard NCT with anti-Her2 therapy [15].

However, while many oncologists would focus on achieving pCR as a landmark of the success of neoadjuvant therapy, more information could be discussed in this setting. For example, in a study by Symmans and colleagues on a pool of 5160 patients from the I-SPY platform, the subdivision of the pathological response using the MD Anderson residual cancer burden (RCB) into four levels of response could provide additional prognostic data to that provided by pCR rates only [16]. This might be much more valuable in our situation with more patients with the large initial disease and luminal subtype (less likely to reach pCR). Such information is crucial when evaluating patients for further adjuvant treatment in patients with residual disease after NCT.

In our low-resource setting, with 78% ORR, neoadjuvant therapy for our young locally advanced patient cohort could allow for significant tumor size reduction (enhancing more breast conservation). Additionally, NCT adds to the long-term survival outcomes by early addressing the expected micrometastatic disease and offers a platform to formulate highly effective and simple prognostication models [17, 18]. This study is one of the few analyses that look into detailed outcomes of Egyptian LABC patients. However, our study is limited by its retrospective nature and depends on a single-center experience. Also, insufficient long-term adjuvant endocrine therapy data leads to its exclusion from the regression analysis. Limitations faced by our patients to complete the full course of therapy were sometimes attributed to delayed reimbursement by other sponsoring entities. Furthermore, larger multicenter studies are needed to validate our findings.

## Conclusions

This retrospective cohort study identifies predictors of clinical and pathological response of breast cancer to NCT. PCR remained an important prognostic factor for survival outcomes in our cohort. Luminal subtypes drive a benefit from NCT with a high ORR, pCR, and breast conservation rates. Reliable clinicopathological parameters as initial tumor stage, high KI-67, optimal chemotherapy, and absence of LVI are helpful to the clinician in limited-resource settings.

## Data Availability

The data that support the findings of this study are available from the corresponding author upon reasonable request.

## Abbreviations

ECE: Extracapsular extension
HER2: Human epidermal receptor 2
HR: Hormone receptor
IDC: Invasive ductal carcinoma
ILC: Invasive lobular carcinoma
LABC: Locally advanced breast cancer
LVI: Lymphovascular invasion
NCT: Neoadjuvant chemotherapy
ORR: Objective response rate
pCR: Pathologic complete response
TNBC: Triple-negative breast cancer
